# A Consensus Model of Homology-Directed Repair Initiated by CRISPR/Cas Activity

**DOI:** 10.3390/ijms22083834

**Published:** 2021-04-07

**Authors:** Kevin Bloh, Natalia Rivera-Torres

**Affiliations:** 1 Gene Editing Institute, Helen F. Graham Cancer Center & Research Institute, ChristianaCare, 4701 Ogletown-Stanton Road, Newark, DE 19710, USA; kbloh@udel.edu; 2 Department of Medical and Molecular Sciences, University of Delaware, Newark, DE 19710, USA

**Keywords:** CRISPR/Cas, homology directed repair, DNA repair, synthesis-dependent strand annealing, gene repair

## Abstract

The mechanism of action of ssODN-directed gene editing has been a topic of discussion within the field of CRISPR gene editing since its inception. Multiple comparable, but distinct, pathways have been discovered for DNA repair both with and without a repair template oligonucleotide. We have previously described the ExACT pathway for oligo-driven DNA repair, which consisted of a two-step DNA synthesis-driven repair catalyzed by the simultaneous binding of the repair oligonucleotide (ssODN) upstream and downstream of the double-strand break. In order to better elucidate the mechanism of ExACT-based repair, we have challenged the assumptions of the pathway with those outlines in other similar non-ssODN-based DNA repair mechanisms. This more comprehensive iteration of the ExACT pathway better described the many different ways where DNA repair can occur in the presence of a repair oligonucleotide after CRISPR cleavage, as well as how these previously distinct pathways can overlap and lead to even more unique repair outcomes.

## 1. Introduction

The mechanism of action and regulation of ssODN-directed gene editing has been a topic of discussion and of great interest for over 20 years. With the rapid development and ease-of-use issue of the latest gene editing technology, CRISPR/Cas has permitted the elucidation and further characterization of the gene editing mechanisms. The CRISPR/Cas system functions naturally in the adaptive immunity pathway of bacteria. Through repurposing, CRISPR/Cas has emerged as the preferred system to catalyze site-specific DNA cleavage [1,2,3,4,5,6]. Cas assembles with a designed gRNA to create a complex that can cut at any desired site in a chromosome. A protospacer adjacent motif (PAM) is required for Cas9 binding, and the target must be upstream of a 5′-NGG-3′ site (with SpCas9) or downstream of 5′-TTTN-3′ (with AsCas12a). The Cas/gRNA complex scans the genome for the PAM sequence. When the Cas/gRNA binds to a PAM, it then locally unwinds the DNA adjacent to the PAM (upstream with Cas9, downstream with Cas12a); if the target region of the gRNA can bind with a perfect match, the Cas nuclease then cuts both strands of DNA [7,8]. Once the CRISPR/Cas complex has created a DSB, cells employ several pathways to repair the damage; these pathways include nonhomologous end joining (NHEJ), microhomology-mediated end joining (MMEJ), and homology-directed repair (HDR) [7,9,10]. NHEJ can occur through canonical NHEJ (C-NHEJ), which ligates the broken ends back together. There is an alternative end joining pathway (alt-NHEJ), in which one strand of the DNA on either side of the break is resected to repair the lesion [10,11]. In traditional DNA repair, NHEJ typically leads to scarless repair, where the DNA ends are religated without losing any sequence information. In the presence of CRISPR constructs, however, this scarless repair leads to recapitulation of the gRNA recognition site, causing recleaving, which shifts the overall repair outcome likelihood significantly in favor of sequence-changing repair outcomes [12]. MMEJ can be utilized to repair broken DNA ends by utilizing patches of microhomology on either end of the break site, leading to specific deletions upon repair [13,14,15,16]. These repair methods are error-prone, meaning that the lesion is repaired imperfectly, resulting in insertions or deletions (indels). If there is a DNA molecule within the local vicinity of the break that shares homology with the region around the double-strand break, then the homologous DNA can be utilized as a template to repair the break via the homology-directed repair (HDR) pathway. Utilization of various CRISPR/Cas systems have greatly increased the pace and lowered the cost of research, expanding the possibilities and allowing for the genetic manipulation of cells and organisms that have historically been difficult to modify [17]. A major challenge in this area is changing a DNA sequence to define gene function through HDR, rather than to delete the gene function via NHEJ/MMEJ, which results in total dysfunction [18,19].

The correction of single base mutations in cells has long been the ultimate goal of gene editing with single stranded DNA oligonucleotides (ssODN) [20,21,22] and its mechanism and regulation has been a greatly discussed topic over the last 15 years [21,22,23,24,25,26,27,28,29,30,31]. With these attempts, a number of elaborate models based on Homology Directed Repair (HDR) have been established [32,33,34]. With the incorporation of CRISPR/Cas9 to the gene editing toolbox, many paired this new technology with ssODNs, leading to higher efficiency of the precise repair of point mutations. By reengineering key reaction components, such as the interaction between CRISPR/Cas9 and the PAM sites [35], chemically modifying the Cas9 [36], ssODN [37], and sgRNA [38] HDR accuracy and efficiency has been significantly increased.

**Figure 1:**
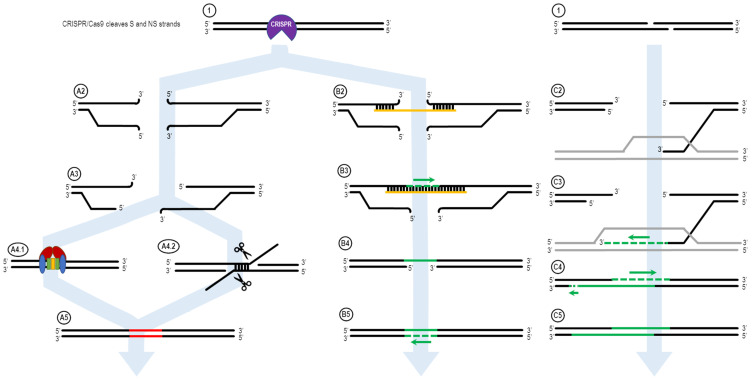
The ExACT DNA repair pathway, as outlined by Rivera-Torres et al. Upon CRISPR-mediated cleavage or breakage of the DNA (**1**), several possible pathways may be undertaken in order to facilitate DNA repair. In the absence of an ssODN oligo, or if the ssODN is not in the local vicinity of the broken DNA ends, the non-oligo repair pathway (**A2**–**A5**) is used. The CRISPR-Cas complex dissociated from the DNA ends (**A2**), and resection of the DNA ends may occur (**A3**). Then, either nonhomologous end joining (NHEJ, **A4.1**) or microhomology-mediated end joining (MMEJ, **A4.2**) occurs to repair the broken DNA ends. This results in an indel in the resulting DNA product, as bases are added or deleted via NHEJ and MMEJ. In the presence of an oligo, the ExACT pathway is undertaken (**B2**–**B5**). The oligo bridges both sides of the broken DNA ends (**B2**) and facilitates synthesis-driven repair on one strand (**B3**). The oligo then dissociated from the repaired DNA strand (**B4**), so the repaired strand can in turn be used as a repair template for a second synthesis-driven repair event on the other DNA strand (**B5**). This results in repair containing added, deleted or altered sequence information from the repair template ssODN. In the presence of a homologous gene, synthesis-dependent strand annealing can occur. One 3′-end on either end of the DNA break interrogates the homologous gene and forms a D-loop (**C2**). The 3′-end is extended via DNA synthesis, expanding the D-loop (**C3**). After a period of extension, the newly-lengthened 3′-end dissociates from the homologous gene, and binds to its complementary sequence on the other end of the DNA break—bridging the gap (**C4**). Further DNA synthesis fills in the remaining bases according to their respective complementary sequences (**C5**).

In 2017, Rivera-Torres et al. [39] introduced the Excision and Corrective Therapy (ExACT) model. This model proposed an overall mechanism by which short oligonucleotides and a CRISPR/Cas9 RNP execute the repair of a point mutation in which the donor DNA acts as a replication template for the repair of the mutant base. In comparison to previously published models [33,40], the original ExACT model was much simpler and was based on the well-accepted and standard model of Double-Strand Break Repair [35,41,42]. In their work, Rivera-Torres et al. demonstrated that it is possible to direct single nucleotide exchange in an efficient manner, but also found indels accompany single base repair. This general concept, outlined in Figure 1, explains the overall reaction of how single-stranded oligonucleotides direct point mutation repair in mammalian cells [22,27,43,44]. The double stranded break in the chromosome induced by CRISPR/Cas activity produces two exposed DNA ends both upstream and downstream of the cut site. These ends are subject to 5′ > 3′ resection, as seen in NHEJ/MMEJ, but these mechanisms are independent of the template-driven repair utilized by ExACT. In the described ExACT model, an ssODN template interrogates both ends of the cut site simultaneously, bridging the gap between ends. This allows for DNA synthesis across the gap, with synthesis occurring in a 5′ to 3′ direction, ending when DNA synthesis catches up to the end of the resected DNA end, leading to dissociation of the synthesis complex and ligation of the newly-synthesized strand into the subsequent DNA strand. After this step of repair, the oligonucleotide is no longer necessary, and must dissociate from the complex in order for the repair to be completed. Once the repair template ssODN is dissociated, the newly-synthesized stretch of DNA on the opposite strand can be used as a template for synthesis-driven fill-in of the remaining bases, followed by varying degrees of resection of the broken ends and extension by the DNA replication machinery. These steps are independent of the desired or intended repair process, HDR or NHEJ. The gap created by the original double-strand break is repaired through the utilization of an exogenous piece of DNA that serves as a source of genetic information and the template for replication activity and gap filling.

The ExACT pathway, in essence, shows some similarity to the synthesis-dependent strand annealing (SDSA) mechanism of DNA repair [44,45,46]. In SDSA, a DNA break is subjected to 5′-to-3′ resection on both ends of the break site, as is seen in MMEJ. In the presence of a homologous gene from a sister chromosome, however, one single-stranded 3′ end of the broken DNA interrogates the sister gene, initiating DNA synthesis and elongation of the 3′ end in accordance with the sequence of the sister gene. Once extended far enough to bridge the DNA break gap, the elongated 3′ end dissociates from the sister gene and re-binds to the opposite end of the break site, allowing the resulting complex to be filled in through DNA synthesis and ligated back into a correctly repaired DNA strand. This differs from the other sister chromosome repair model, the Double-Holliday Junction Model [47], as in SDSA where there are no DNA strands that move from one chromosome to another. In SDSA, there was no crossing-over event seen, while the Double-Holliday Junction Model requires crossing over for complete DNA repair [48]. In common discussions related to CRISPR gene editing, SDSA is invoked as a repair mechanism for oligonucleotide-driven repair. The mechanism through which oligonucleotide-driven repair would occur, however, does not fit the traditional description of SDSA. In SDSA, the resected DNA end of a broken DNA strand interrogates an unbroken dsDNA template from a sister chromosome. In oligonucleotide-driven HDR, however, the oligo interrogates the broken DNA ends instead. This difference, while slight, can lead to multiple different downstream effects that are unique to each mechanism. In SDSA, for instance, incorporation of the repair template directly into the broken DNA is impossible, as no crossing over events occur, leading to ligation between the broken DNA and the repair template.

In relation to CRISPR gene editing repair, the distinction between semi-conservative and conservative DNA repair can be seen when editing members of homologous gene families. In multiple papers by several labs studying CRISPR editing in sickle cell anemia, targeting the hemoglobin beta gene (HBB) with a CRISPR/Cas construct led, in a small number of cases, to a conversion of a segment of the HBB gene to that of hemoglobin delta (HBD), effectively creating a novel HBB/HBD chimeric gene [32,49,50]. When the hemoglobin delta gene was sequenced in these HBB-HBD converted cells, the HBD conversion was found to be non-reciprocated, with no segment of the HBB sequence present within the HBD gene. This points to a fully conservative method of synthesis-directed gene repair and excludes the semi-conservative Double Holliday Junction Model from consideration as a potential pathway utilized to generate the given data. While DHJ remains an established and known pathway for some forms of gene repair and conversion, there remains no evidence of this particular pathway occurring in CRISPR-directed gene editing. Furthermore, when examining repair mechanisms utilized in the presence of a ssODN repair template, DHJ becomes impossible as a repair mechanism, as the short length of the ssODN would result in chromosome truncation if semi-conservative gene repair did take place. 

## 2. ExACT Pathway vs. SDSA DNA Repair Mechanism

Figure 1 shows the ExACT pathway. As explained by Rivera-Torres et al. [39], the ExACT pathway requires, as a prerequisite to repair, CRISPR-cleaved DNA ends and a repair template with a specific genetic change. Upon cleavage by the CRISPR-Cas complex, multiple pathways can occur concurrently and simultaneously at the broken DNA ends. In the absence of a homology donor, or if the donor is not in the local area of the cut DNA ends, nonhomologous end joining (NHEJ) and microhomology-mediated end joining (MMEJ) can occur. These two lossy DNA repair pathways both take advantage of DNA resection at the cut site in order to open the DNA ends for repair. In the presence of Ku70/80 stabilizing the DNA ends (Figure 1, A4.1), NHEJ occurs to seal the DNA ends with minimal sequence loss at the cut site [11,51]. Indels that occur through NHEJ are typically small (>30 bp) and random; indels across a population of cut DNA ends, both in vitro and in vivo, will have a plurality of indels at the cut site, rather than 1–3 major ones. 

In many cases, however, MMEJ is readily seen at a cut site, even with just the slightest microhomology around the cut site. During MMEJ, single-stranded resection of the broken DNA ends reveal patches of DNA both upstream and downstream of the cut site that incidentally share sequence homology. These areas of microhomology associate with each other, leading to temporary stabilization of the broken DNA ends (Figure 1, A4.2). The degree of stabilization is dependent on the extent of the microhomology, but as can be seen in Figure 4D, as little as 4 bp is enough to establish strong enough microhomology to overcome NHEJ as the predominant indel source. 

When an ssDNA repair template is present in the reaction, however, a separate pathway is taken. This pathway, displayed on the right side of Figure 1, is the ExACT pathway. The ssDNA repair template bridges the DNA break on one strand and associates with the perfect-match sequences both upstream and downstream of the cut site. Depending on the speed of DNA end resection and the size of the DNA oligo, this process may curtail the resection process by bridging the gap further downstream of any resected bases. Once the cut site is stabilized with the oligonucleotide, the missing bases across the cut site are filled in via DNA polymerase, using the repair oligo as a negative-strand guide. This allows any changes intentionally incorporated into the repair template to be copied into the repaired DNA strand, including base changes, insertions, or deletions (B3). Once the strand complementary to the repair template is fully repaired and ligated, the oligonucleotide dissociates from the junction, leaving a single-strand gap around what was once the CRISPR cleavage site (B4). This single-stranded gap is then filled in by DNA polymerase using the previously repaired DNA sequence as a guide (B5). This results in a cleanly repaired DNA template with no need for incorporation of the ssDNA oligo to facilitate repair.

The mechanism of the ExACT pathway bears many similarities in concept to the Synthesis-Dependent Strand Annealing pathway. Both allow for DNA repair to occur without disruption of the reference DNA sequence, as compared to the Double Holliday Junction Model, which leads to a crossover event between the repair reference DNA sequence and the cut DNA ends. One major mechanistic difference between the two pathways, however, is the simultaneous binding of both open DNA ends in the ExACT model. In SDSA, the repair template, a double-stranded DNA homolog, interrogates a single broken DNA end and allows for polymerization from that end to extend the 3′-end of the cut. This extended 3′-end then bridges the break site, binding to its complement on the opposite end and allowing for the filling in of any gaps in the sequence through polymerase activity [52]. The two-step polymerization process employed in SDSA differs from the single-step bridging of the gap outlined in the originally published ExACT manuscript [39].

This distinction between a single gap-bridging event and a two-step polymerization process is a subtle difference, and further research into the physiochemical structure of these D-loop formations will need to be performed in order to empirically ascertain which mechanism, if either, are being employed in in vivo gene editing reactions. Further testing would then have to be performed in order to verify that the structures formed within the in vitro system replicate those seen in live cells. Regardless, the presence of a one-step or two-step gap-bridging event would be able to be seen in the secondary indels observed in non-perfect HDR events. These events are typically much more rare in gene editing reactions, as they require one of these structures to resolve incorrectly. Due to how rare these events are in in vivo gene editing, it is likely that, even if discovered, these secondary incorrect recombinations would also be found in the appropriate quantities to evidence the necessary events in order to distinguish mechanisms as subtly different as a one-step or two-step gap bridging event taking place. The overall rarity of these events being recordable in in vivo editing, in addition to the massive amount of targeting, clonally expanding, and sequencing that would be necessary to generate such datasets would prevent any conclusive statements about the formation of these atypical repair outcomes. However, using the in vitro system, such specific hypotheses can be tested with ease.

The in vitro system has served to examine the molecular mechanism of CRISPR-mediated gene repair in a tightly-controlled, optimized system [53,54]. In this system, the CRISPR-Cas RNP complex provides the double-stranded cleavage and a mammalian cell-free extract provides the enzymatic activity to promote activation of the DNA repair pathways in the presence of a donor DNA template. Briefly, a CRISPR cleavage reaction is performed on a plasmid construct containing a gene of interest (in this case, LacZ). The linearized plasmid, cleaved at the CRISPR target site, is then re-circularized using a cell-free extract isolated from live cells, containing all of the necessary components of DNA repair, at the proper concentrations and availabilities. This effectively mimics DNA repair seen in a live cell, without having to account for many of the confounding factors commonly seen in in vivo gene editing, such as nuclear uptake and low DNA cleavage efficiencies [53]. 

**Figure 2:**
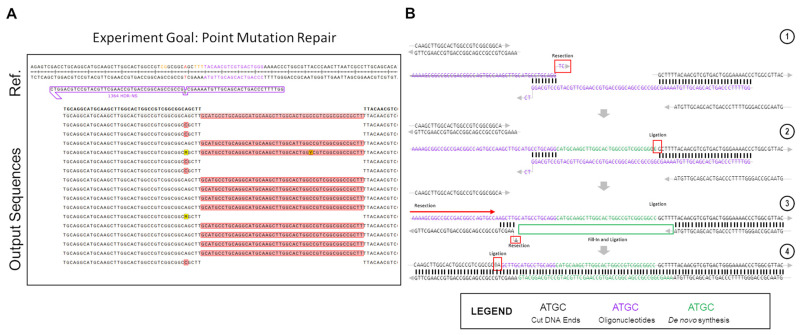
Atypical repair outcomes caused by ssODN dimerization in in vitro gene editing. The partial outcome data of one gene editing reaction, carried out using Cas12a in the in vitro system, are shown here. (**A**) The experimental results. The LacZ gene was repaired with a semi-symmetrical oligo, with one 40 bp and one 29 bp homology arm, with a single base repair (A > C) in the middle. (**B**) Depiction of the pathway of repair that led to the 48 bp insertions seen in the experimental output. (**1**) The dimerized ssODN (purple) associated with the cut DNA ends (black) in order to facilitate the generation of the products seen. (**2**) The dimerized ssODN created a makeshift gap that allowed for DNA synthesis to fill in according to the template (green). Ligation occurs after the green bases are synthesized, incorporating the actual oligo into the top strand of the downstream end as a long 5′-overhang. (**3**) After ligation, the unincorporated ssODN dissociates from the complex, and the newly-extended 5′-overhang associates with a 5bp stretch of microhomology on the upstream end of the cut site. The 5′-end upstream of this microhomology patch is resected or cleaved, as well as the single mismatched base on the exposed 5′-end of the bottom strand. (**4**) Ligation occurs after resection on the top strand, leading to a complete top strand. The bottom strand, meanwhile, is filled in via synthesis, using the top strand as a template.

Figure 2 shows the partial outcomes of an experiment that was carried out within the in vitro system. The goal of the experiment was to test single base repair competencies using an ssODNs. Due to the highly optimized nature of these reactions in vitro, we were only able to screen sequences that showed editing through the bacterial LacZ readout [53].

A significant portion of the non-perfect repair outcomes in Figure 2A consist of 48 bp insertions of the same DNA sequence. This sequence is consistent with the repair oligo, but in the opposite orientation, as would be expected if the oligo was undergoing normal DNA repair. Instead, what appears to be happening in this case is an in-line incorporation of the oligo against its normal orientation, with the negative sense-strand oligo being incorporated in-line with the sense-strand gene sequence. The high rate at which this specific indel was seen in the outcome points to a highly-reproducible event being the culprit for the peculiar recombination. While accounting for many different potential causes for the indel, most, while still possible, lacked the unique determined repair outcome, or implied the possibility of several varying outcomes, which were not seen in the results, and as such were eliminated from the contention as the cause for these repairs. Upon full examination of the potential causes for such a replicable, but atypical insertion, it was eventually realized that an ssODN dimerization had occurred, as displayed in step 1 of Figure 2B. Using Integrated DNA Technologies OligoAnalyzer tool, the delta-G of the dimerized oligo was found to be −16.38 Kcal/mol, with 8 bp of homology on the 3′-ends of the ssODN. There was another product with a more energetically favorable delta-G, at −22.78 kCal/mol, but this dimer created 3′ overhangs, which would prevent the dimer from associating with the free DNA ends left after Cas12a cleavage (Figure S1). 

This atypical repair outcome provides an interesting example of potential DNA repair after CRISPR-Cas cleavage, as in this situation, it appears as if both ExACT repair and SDSA-like repair is occurring sequentially within these repair events. The proposed pathway for the atypical repair is seen in Figure 2B. First, the oligo associates with the downstream DNA end, while simultaneously dimerizing with itself at the previously described 8bp stretch. This complex is incredibly similar to the one-step gap-bridging event outlined in the ExACT pathway, but with a dimerized oligo being used in place of the top strand of the DNA upstream of the cut site. The final two bases of the ssODN, which do not match complimentarily, are removed via flap endonucleases or the limited 3′>5′ resection seen in DSB repair complexes. Using the properly oriented ssODN as a template, the gap is then filled in via DNA synthesis, with the end ligated to the downstream plasmid DNA end. Once this ligation occurs, the properly oriented oligonucleotide is not necessary for further repair, and in fact may hinder further repair if it remains bound. 

This resulting repair outcome, however, still does not lead to recircularization of the plasmid, which had to have occurred for these DNA sequences to be amplified. This repair outcome alone would have merely led to an extended linearized plasmid, which would not have been able to be amplified with the utilized primers. Thus, it was determined that, after this initial repair event, a second synthesis-driven repair would have had to take place in order to properly re-circularize the plasmid. Due to the incorporation of the improperly-oriented oligo due to dimerization, the DNA ends at the cut site are changed, with the downstream end now containing a long 5′ overhang that happens to match a 5 bp microhomology patch upstream of the cut site. This complex, displayed in Step 3 of Figure 2B, aligns more closely with the repair complex seen in the SDSA-like repair. There are still discrepancies as this complex is formed with a long 5′ overhang, as opposed to a long 3′ overhang. However, the end product is similar, as the top strand, after resection of the unmatched bases, and can simply be ligated to the top strand of the plasmid DNA without the need for further DNA synthesis. This type of repair, which occurs second, appears to be distinct from either traditional SDSA repair or ExACT, but bears similarities to SDSA. Another repair pathway example found in a different experiment that was created by dimerizing repair ssODNs is included in Supplemental Figure S2. It should be made clear, again, that this dimerization of the oligo is not thought to be the mechanism by which the ExACT pathway repairs DNA ends. The outcomes shown in these data are repair outcomes that are enriched in the population due to unforeseen problems with repair template design (i.e., the higher energy favorability of oligo dimerization compared to repair homology). However, these imprecise outcomes still indicate that both single-event gap bridging and multi-stage repair can occur, which further indicates that the ExACT model, as described previously, is incomplete in its description of DNA repair and must be updated to include these SDSA-like repair mechanisms. 

## 3. The gRNA-Dependent Strand Bias Seen In Vitro Is not Due to ExACT or SDSA DNA Repair Mechanisms

The secondary HDR-mediated indels seen in the in vitro system reactions point to multiple disparate gene repair pathways working independently in response to different post-cleavage repair environments. These different repair environments may be influenced by many unpredictable factors within in vitro and in vivo, such as the local availability of repair templates at the DNA break site or the resulting growth advantage or disadvantage of a specific gene editing product. Several other factors, however, can be adequately predicted during the design stage of a gene editing reaction. Free energy calculations of repair templates performed before the editing reactions shown in Figure 2 could have potentially predicted, or at least suggested, the complex repair outcomes seen in the data. Likewise, one major factor present in both HDR and non-HDR CRISPR gene editing is strand bias [53,54,55,56]. 

As described before by Sansbury et al., strand bias is seen in the in vitro system [54,56]. This discrepancy in HDR-mediated editing efficiency between S-Strand and NS-strand repair templates is seen whenever the two are directly compared, in both Cas9 and Cas12a-directed editing reactions [54]. This bias has been previously described as potentially related to both the orientation of the gRNA and resulting structure of the DNA break, and the orientation of the gene itself and whether the leading or lagging strand in DNA synthesis was displaced by the repair ssODN [22,57,58]. Using the in vitro system and expanding the scope of editing sites beyond those shown in previous works, we can make a strong case that strand bias in Cas12a-mediated gene editing is much more heavily influenced by gRNA orientation than by gene orientation. 

**Figure 3:**
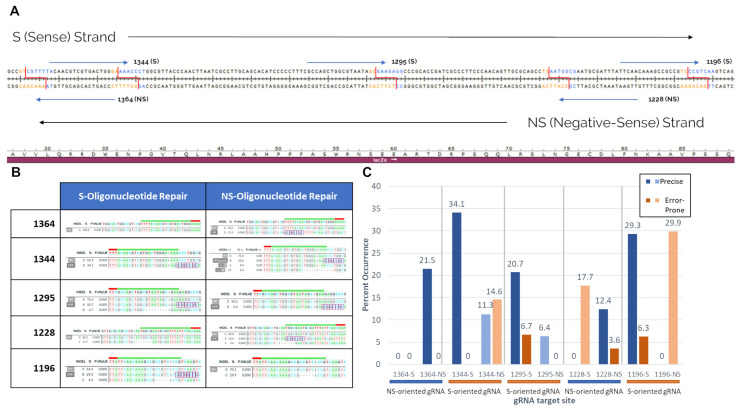
Secondary non-ExACT products seen in ssODN-containing reactions. (**A**) Schematic of the LacZ site tested in the in vitro system. Five total sites were analyzed (blue arrows represent gRNA), with the cleavage sites highlighted with red lines. Each cut site was repaired with one of two symmetric ssODNs identical to the sense or negative-sense strand, containing an 8 bp insertion directly at the specific cut site. The bases in yellow and the bases in blue represent the DNA ends resulting from each gRNA cut upstream and downstream, respectively. (**B**) Resultant repair outcomes for each site, with each ssODN utilized at that site. Deconvolution and alignment of Sanger sequencing products of each in vitro product are displayed, with inserted bases highlighted with purple boxes and deleted bases shown as dashes. Relative percentages of each product are also shown. (**C**) Quantification of non-HDR indel percentages. For each of the five cleavage sites, the rates of precise (ExACT-driven) and error-prone (MMEJ/NHEJ-driven) products are shown in blue and orange, respectively. For reactions that contained gRNAs and oligos in the same orientation, darker colors are used, while reactions that used gRNAs and oligos of the opposite orientation are displayed with lighter colors.

Figure 3 displays pairwise comparisons of S-strand and NS-strand gene editing reactions carried out with the in vitro system. Reactions were carried out at five sites within the LacZ gene, with the results of gene repair using either an S-Strand or NS-Strand ssODN repair template being compared. The repair template orientation was labeled, reflecting its orientation to the gene, rather than to the gRNA. Each HDR template contained 36 bp homology arms on either side of the cut site, along with an 8 bp NotI restriction site in the middle. The editing products, determined by Sanger sequencing, are seen in Figure 3B. As can be seen in all cases, the only HDR-mediated gene editing product seen is the perfect NotI insertion. 

The direct comparisons between the gRNA orientation and strand bias can be seen in Figure 3C. The darker-colored bars in each section correspond to the repair outcomes of the ssODN template that matches the gRNA’s orientation, while the lighter colored bars correspond to the ssODN template that is complementary to the gRNA. As can be seen across all five sites, the ssODN that matched the polarity of the gRNA was determined to have a higher overall HDR efficiency, typically by a large margin. In fact, not only did HDR efficiencies use more ssODNs, which matched the gRNA polarity, but secondary MMEJ products also used less. This corresponds to both what we have shown previously in the in vitro system with Cas12a, as well as what has been seen in in vivo Cas9 gene editing [55]. 

Interestingly, this HDR strand bias does not appear to follow the structure of the cut DNA ends, as Cas12a leaves 5bp 5′-overhangs regardless of the orientation of the gRNA. Both the 1364 and 1344 sites, for example, provide 5 bp 5′-overhangs at nearly the same site. However, the NS-gRNA 1364 shows much higher HDR efficiency with the NS-oriented ssODN, while the S-gRNA 1344 site shows higher HDR efficiency with the S-oriented oligo. This strand bias persists even when accounting for the potential perseverance of the Cas complex bound to the DNA ends after cleavage (Figure S2). The post-cleavage binding of Cas12a is not a major contributing factor to strand bias. As stated in previous publications, the in vitro system separates the DNA cleavage and recircularization steps, meaning that the Cas RNP are fully dissociated from the DNA, and any residual gRNA is removed from the reaction before any of the DNA repair enzymes present in the cell-free extract are ever exposed to the broken DNA ends. Taken together with the gRNA-dependent strand bias, these data suggest that another factor is responsible for this strand bias, and that previously-assumed factors, such as gRNA-mediated oligo chelation and individual oligo binding affinities at the cut site [59,60], are not the predominant contributor to strand bias in these reactions.

## 4. Secondary Indel Patterns Caused by Other Pathways Are Caused Alongside ExACT Products

It is known that error-prone DNA repair, such as NHEJ and MMEJ, as well as precise repair methods such as HDR and ExACT, can take place simultaneously in a gene editing reaction, even on the same cut DNA ends [54]. This is recapitulated in the in vitro system. In Figure 3B, secondary non-exact products are seen at several of the sites. These secondary non-exact products occur independently of whether ExACT repair is seen or not, but also appear to be dictated by the presence or absence of patches of microhomology before and after the cut site. 

These secondary products are likely caused by microhomology-mediated end joining, as each indel contains patches of microhomology before and after the cut site that match the length and position of the determined indels. In order to confirm that these secondary products were caused by MMEJ, we performed several in vitro reactions without any repair templates present. In these reactions, the only products that would be formed would be the result of non-precise gene repair mechanisms, such as MMEJ and NHEJ. We examined the 1364 site, which is the most well-characterized site in our previous work, and the 1344 site, which has a near identical gRNA site to 1364, but with the opposite polarity. Finally, we chose the 1228 site, as it showed strong secondary error-prone products in both S and NS ssODN reactions. 

**Figure 4:**
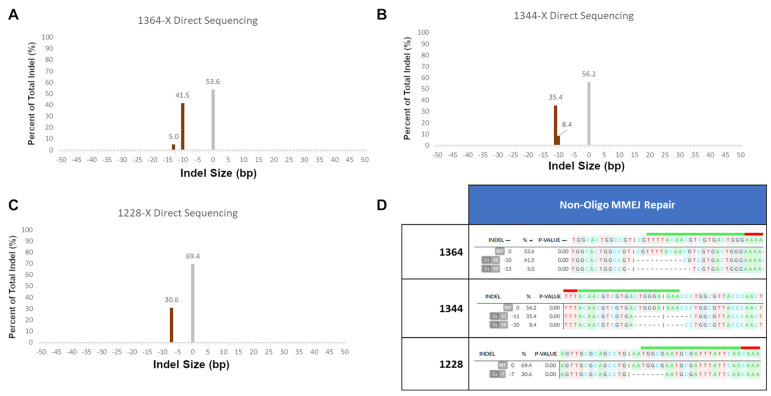
Non-Exact repair outcomes in reactions containing no repair ssODN. Gene repair reactions were performed using the in vitro system in the absence of and ssODN repair template, to determine whether or not the secondary products seen in Figure 3 were influenced by the presence of a repair template. Unedited sequences are displayed in gray and edited sequences are shown in dark orange. (**A**) Indel products seen when targeting the 1364 LacZ site. (**B**) Indel products seen when targeting the 1344 LacZ site. (**C**) Indel products seen when targeting the 1228 LacZ site. (**D**) Specific resultant repair outcomes seen in the three non-ssODN reactions, deconvoluted and aligned using DECODR [61]. The alignment of the gRNA is shown as a green bar at the top of the reference sequence (with a red end for the PAM site), and the specific cut site on the S-strand of the DNA is shown as a pipe (|). Deleted bases are represented by dashes.

Figure 4 shows the indel products seen in these three experiments. Unexpectedly, the 1364 site showed the highest rate of error-prone repair of all three experiments, with over almost 50% of all sequences being caused by error-prone repair. The two products seen in the analysis appear to both be caused by microhomology-mediated end joining; a 6 bp CGTCGT repeat is present both at the cut site and 10 bp downstream. These CGT repeats are likely responsible for both the 10 bp and 13 bp deletions seen in the output. These microhomology patches and their subsequent products are also visualized in Supplemental Figure S3.

A similar MMEJ pattern is seen at the 1344 site. In this case, the 11 bp deletion that makes up a majority of the indel total in the non-ssODN reaction is identical to the secondary product seen in Figure 3B. In addition to the −11 bp product, a similar −10 bp product is seen as well. Both of these appear to be caused by the 4 bp CTGG repeats 6 bp upstream and 5 bp downstream of the cut site. Finally, at the 1228 site, the indels seen are again identical to the ones seen in Figure 3B. The indel in this case appears to be caused by a 5 bp GAATG repeat 1bp upstream and 7 bp downstream of the cut site. 

In all three cases shown in Figure 4, it appears as if microhomology-mediated end joining is responsible for the indels at each of these sites. This is to be expected, as all three sites have patches of microhomology in relatively proximity to the cleavage site. All three of these sites, in the presence of a repair oligonucleotide, show depressed levels of error-prone repair, even when, as seen in 1228 with the S-oligo, the ExACT-mediated precise repair did not occur; in this case, error-prone repair was 17.7% with oligo compared to 30.6% without. Overall, the only error-prone repair product not accounted for from Figure 3B is a 1 bp deletion at the cut site in 1344 repaired with the NS ssODN (Figure 3B, second right panel). Due to the small size of the indel, it could be speculated that it is the product of NHEJ. However, this cannot be confirmed. 

It is apparent that the secondary products seen, even in oligonucleotide-driven repair reactions, are caused by MMEJ and NHEJ, independently acting on the ExACT pathway. However, from the data shown, no examples can be seen of ExACT and MMEJ/NHEJ acting on the same DNA ends. Analyzing editing outcomes with and without repair oligonucleotides via a higher-fidelity sequencing method such as targeted amplicon NGS could find these much rarer precise/error-prone hybrid repair outcomes. Preliminary research into these methods has shown hybrid repair outcomes at a frequency of less than 1:1000 (data not shown). 

## 5. Synthesizing the ExACT and SDSA Models for Gene Repair

While it is apparent that the SDSA and ExACT pathways are similar in function, broken DNA ends are repaired from a repair template without incorporating the template into the final repaired product. However, the intricacies of each pathway differ in ways that can potentially cause different forms of imprecise repair outcomes. The flipped and incorporated repair outcomes seen in Figure 2 indicates that a single cut-site bridging step takes place, with the repair oligo or dimerized oligos binding to both sides of the cut site before polymerase-based extension and fill-in takes place. However, this pathway alone is not sufficient to explain the extent of editing products seen. ExACT-mediated repair alone could not have generated the products seen in Figure 2, and so the model must be updated to account for these new data. 

**Figure 5:**
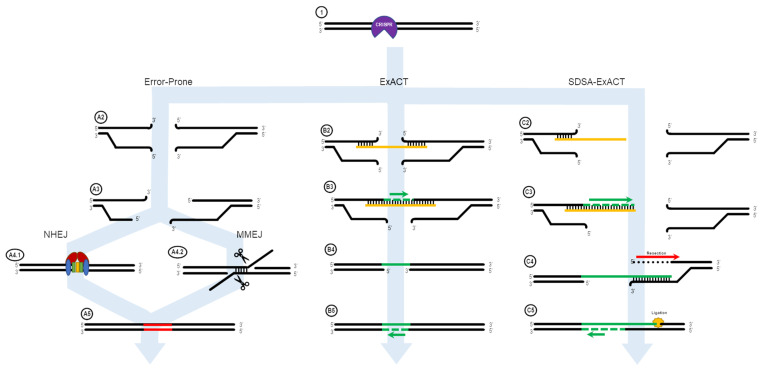
A more comprehensive ExACT pathway. This redesigned ExACT pathway incorporates the two-step gap bridging mechanism and depicts the possibility for overlap between the multiple branching arms of the pathway. After CRISPR-Cas cleavage (**1**), the complex dissociates from the DNA. In the local absence of ssODN repair templates, the DNA ends are resected (**A3**). NHEJ and MMEJ occur to repair and relegate the broken DNA ends (**A4.1** and **A4.2**), resulting in an error-prone final product containing indels. In the presence and accessibility of a repair ssODN, the oligo will either bind to both or just a single end of the broken DNA (**B2** and **C2**, respectively). If both ends are stabilized by the ssODN, DNA synthesis and ligation to the opposite end are performed concurrently via DNA synthesis (**B3**). Once the oligo the dissociates (**B4**), the repaired strand can then be used as a template for a second synthesis step, repairing the other strand (**B5**). If only a single end associates with the ssODN, synthesis occurs from the 3′ end of the broken DNA, extending it using the ssODN as a template (**C3**). This extended end can then, once the oligo dissociates, bind to the opposite end of the cut site and bridge the DNA break. At this time or previously, the S strand of the downstream end is resected in a 5′ > 3′ direction (**C4**). Finally, the now-bridged DNA can be repaired via synthesis using the newly-synthesized upstream sequence as a template via a second round of synthesis. The final DNA break is then repaired wither via simple ligation or extension and ligation of the S strand sequence (**C5**).

It therefore appears that both the gap-bridging ExACT and the two-step SDSA-like repair are potential repair pathways that can occur in different oligo-driven gene editing reactions. There is, when looking at all established conservative DNA synthesis-dependent repair models (SDSA, ExACT, MMEJ), no single DNA repair mechanism that can be seen as solely responsible for all potential CRISPR-mediated DNA repair outcomes. As such, it is necessary, given the evidence, to revisit the initial ExACT mechanism and to redesign the model to include these other potential pathways. This approach allows for the expansion of a single model in order to include real outlier data that is not adequately explained through a single other model. The result, as seen in Figure 5, is a more complex, though more complete, model. 

Figure 5 displays a more comprehensive design for DNA repair using single-stranded oligonucleotides that incorporate all these potential avenues. The three classifications of repair are categorized into: “error-prone,” containing DNA repair mechanisms that do not involve oligonucleotide contribution such as NHEJ and MMEJ; “ExACT,” following the previously described ExACT pathway; and “SDSA-ExACT,” following the newly synthesized SDSA and ExACT pathways, which utilizes a two-step DNA-bridging approach that falls more in line with what has been described in SDSA.

Designing experiments to further test the efficiency and likelihood of each of these pathways can be done within the in vitro system. Designing targeting reactions for synthetic gene constructs allows for complete control over the sequence of the DNA target; for example, designing an experiment with a known patch of microhomology between the far end of the oligonucleotide and the cut site may shift the repair outcomes in order to better characterize the new SDSA-ExACT pathway. With this experimental design, repair outcomes would likely include both perfect repair and duplicated DNA sequences containing the stretch of bases between the microhomology site and the cut site. The extent to which target DNA sites can be customized within the in vitro system makes it much more approachable as an option, compared to searching for a site within the genome where such an event could serendipitously be found. 

It will be important, however, to fully examine how the prevalence of one pathway or another can differ when moving from in vitro experimentation to live-cell targeting. A difference in mechanism between cell-free extract and live cells would need to be accounted for. It will also be very important to keep in mind that these different pathways will be utilized to different extents in different cell types. Cancer cell lines, for example, typically have fewer inhibitors and checkpoints on DNA repair mechanisms [62]. This would likely be reflected in the rate of ExACT or SDSA from one cell line to another. All of the in vitro experiments carried out in this publication utilize a cell-free extract derived from HEK293 cells. We would expect our outcomes would differ a great deal if a different cell-free extract, like K562 or even primary cells, were utilized. 

Though it was not included in the final DNA repair figure, the prevalence of the dimer-based insertions is something that carries potential therapeutic implications. For the original purposes of all the experiments outlined in this paper, these products were deemed imprecise secondary products for the originally intended perfect repairs. However, these oligo dimers allowed for repair at a relatively high rate with just 5–6 bp of microhomology at the upstream DNA end in both experiments. Refining and better elucidating the intricacies of such an approach could lead to an interesting new avenue for high-fidelity and robust gene editing. 

## Data Availability

All data is presented through the manuscript; no databases were utilized.

## Supplementary Materials

The following are available online at https://www.mdpi.com/article/10.3390/ijms22083834/s1, Figure S1: Analysis and pathway of atypical repair resulting in ssODN dimer-driven repair outcomes, Figure S2: Persistence of strand bias within in vitro gene editing reactions in response to post-cleavage Cas12a degradation. Figure S3: Suggested patches of microhomology responsible or non-HDR indel events exhibited in Figure 3 and Figure 4.
