# Supplementary material for: A Consensus Model of Homology-Directed Repair Initiated by CRISPR/Cas Activity
Source: Int J Mol Sci. 2021 Apr 7;22(8):3834. doi: 10.3390/ijms22083834 (PMC8067812; doi:10.3390/ijms22083834)
Supplement: Supplementary file 1 [file ijms-22-03834-s001.pdf]

A Consensus Model of Homology-Directed Repair Initiated by CRISPR/Cas Activity

Kevin Bloh [1], Natalia Rivera-Torres [1,2*]

[1] Affiliation 1; Gene Editing Institute, Helen F. Graham Cancer Center & Research Institute, Christiana Care Health System, 4701 OgletownStanton Road, Newark, Delaware 19710

[2] Affiliation 2; Department of Medical and Molecular Sciences, University of Delaware, Newark, Delaware 19710

* Correspondence: Natalia.riveratorres@christianacare.org; Tel.: 1-302-623-4754

SUPPLEMENTAL MATERIAL

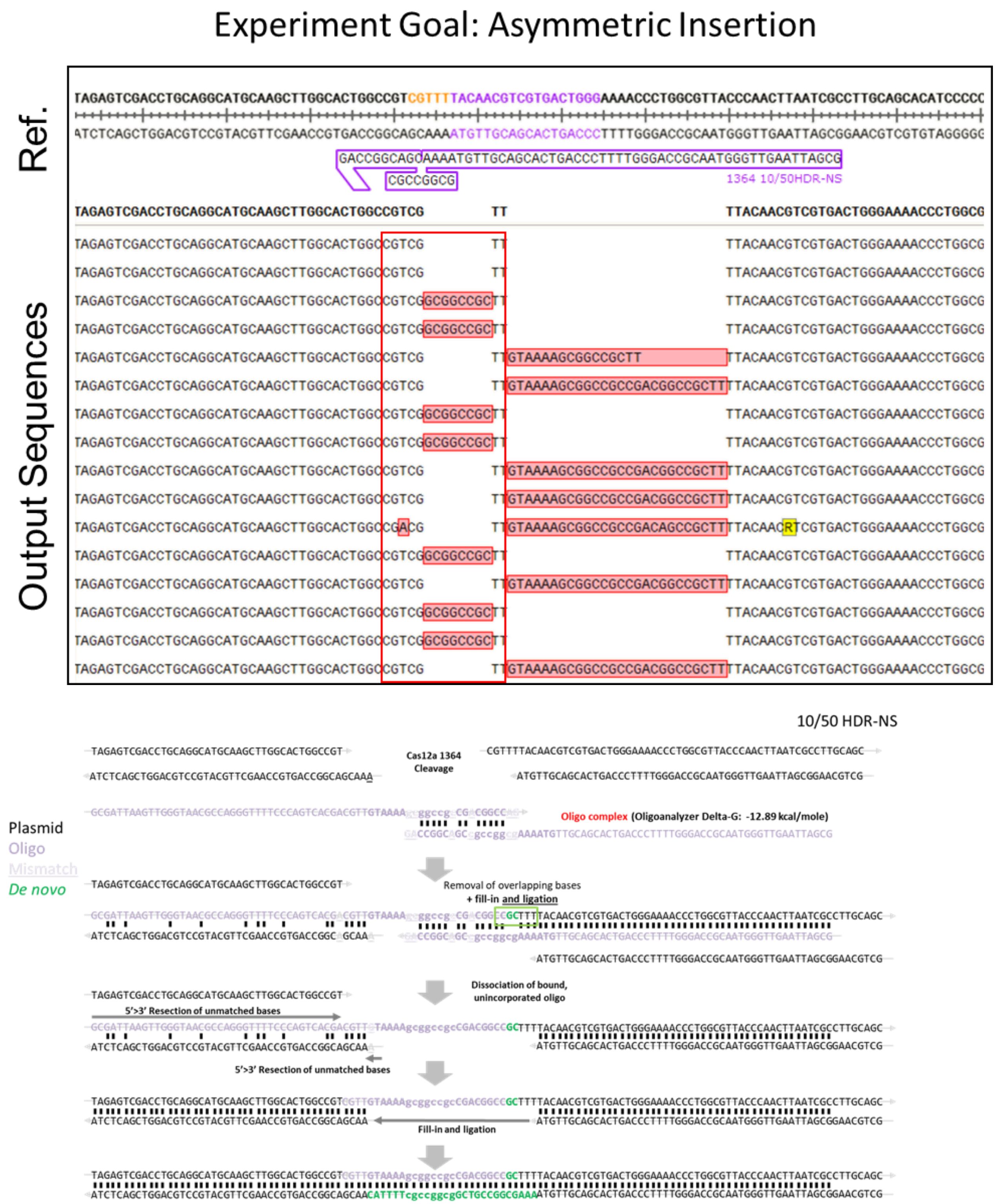

Supplemental Figure 1: Analysis and pathway of atypical repair resulting in ssODN dimer-driven repair outcomes.

Sanger 1344-S/NS Reaction Parameters Comparison

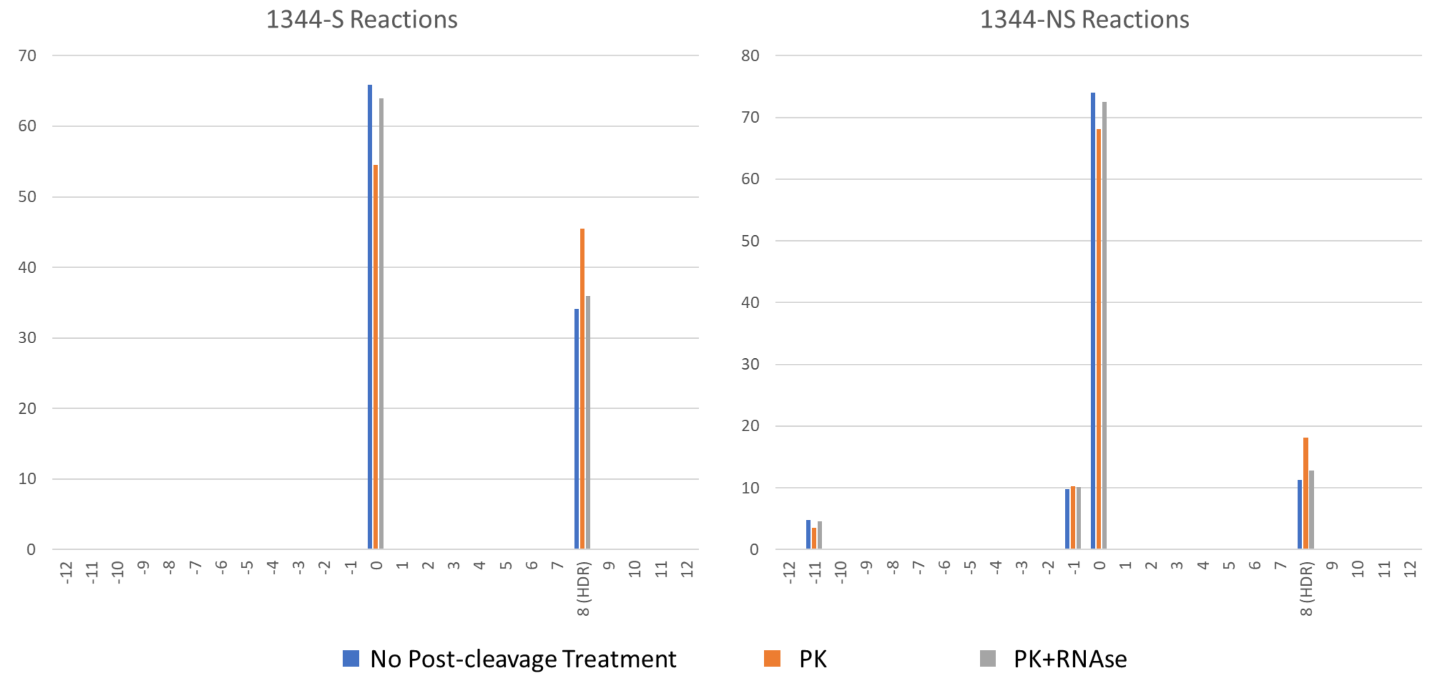

Supplemental Figure 2: Persistence of Strand Bias within *In Vitro* gene editing reactions in response to post-cleavage Cas12a degradation

1364 Microhomology Sites

5TCTAGAGTCGACCTGCAGGCATGCAAGCTTGGCACTGGCCGT3 5CGTTTTACAACGTCGTGACTGGGAAAACCCTGGCGTTACCCAACTTA3

3AGATCTCAGCTGGACGTCCGTACGTTCGAACCGTGACCGGCAGCAAA5 3ATGTTGCAGCACTGACCCTTTTGGGACCGCAATGGGTTGAAT5

5TCTAGAGTCGACCTGCAGGCATGCAAGCTTGGCACTGGCCGTCGTCGTGACTGGGAAAACCCTGGCGTTACCCAACTTA3

3AGATCTCAGCTGGACGTCCGTACGTTCGAACCGTGACCGGCAGCAGCACTGACCCTTTTGGGACCGCAATGGGTTGAAT5

OR

5TCTAGAGTCGACCTGCAGGCATGCAAGCTTGGCACTGGCCGTCGTGACTGGGAAAACCCTGGCGTTACCCAACTTA3

3AGATCTCAGCTGGACGTCCGTACGTTCGAACCGTGACCGGCAGCACTGACCCTTTTGGGACCGCAATGGGTTGAAT5

Expected main MMEJ outcome: -10, -13bp

1344 Microhomology Sites

5AAGCTTGGCACTGGCCGTCGTTTTACAACGTCGTGACTGGGA3 5AAACCCTGGCGTTACCCAACTTAATCGCCTTGCAGCACATCCCCCTT3

3TTCGAACCGTGACCGGCAGCAAAATGTTGCAGCACTGACCCTTTTGG5 3GACCGCAATGGGTTGAATTAGCGGAACGTCGTGTAGGGGGAA5

5AAGCTTGGCACTGGCCGTCGTTTTACAACGTCGTGACTGGCGTTACCCAACTTAATCGCCTTGCAGCACATCCCCCTT3

3TTCGAACCGTGACCGGCAGCAAAATGTTGCAGCACTGACCGCAATGGGTTGAATTAGCGGAACGTCGTGTAGGGGGAA5

Expected main MMEJ outcome: -11bp

1228 Microhomology Sites

5GAGGCCCGCACCGATCGCCCTTCCCAACAGTTGCGCAGCCTG3 5AATGGCGAATGCGATTTATTCAACAAAGCCGCCGTCCCGTCAAGTCA3

3CTCCGGGCGTGGCTAGCGGGAAGGGTTGTCAACGCGTCGGACTTACC5 3GCTTACGCTAAATAAGTTGTTTCGGCGGCAGGGCAGTTCAGT5

5GAGGCCCGCACCGATCGCCCTTCCCAACAGTTGCGCAGCCTGAATGCGATTTATTCAACAAAGCCGCCGTCCCGTCAAGTCA3

3CTCCGGGCGTGGCTAGCGGGAAGGGTTGTCAACGCGTCGGACTTACGCTAAATAAGTTGTTTCGGCGGCAGGGCAGTTCAGT5

Expected main MMEJ outcome: -7bp

Supplementary Figure 3: Suggested patches of microhomology responsible or non-HDR indel events exhibited in Figures 3 and 4.
